# Decoupling of soil carbon and nitrogen turnover partly explains increased net ecosystem production in response to nitrogen fertilization

**DOI:** 10.1038/srep46286

**Published:** 2017-04-13

**Authors:** Emad Ehtesham, Per Bengtson

**Affiliations:** 1 Department of Biology – Microbial Ecology, Lund University, Lund, Sweden

## Abstract

During the last decade there has been an ongoing controversy regarding the extent to which nitrogen fertilization can increase carbon sequestration and net ecosystem production in forest ecosystems. The debate is complicated by the fact that increased nitrogen availability caused by nitrogen deposition has coincided with increasing atmospheric carbon dioxide concentrations. The latter could further stimulate primary production but also result in increased allocation of carbon to root exudates, which could potentially ‘prime’ the decomposition of soil organic matter. Here we show that increased input of labile carbon to forest soil caused a decoupling of soil carbon and nitrogen cycling, which was manifested as a reduction in respiration of soil organic matter that coincided with a substantial increase in gross nitrogen mineralization. An estimate of the magnitude of the effect demonstrates that the decoupling could potentially result in an increase in net ecosystem production by up to 51 kg C ha^−1^ day^−1^ in nitrogen fertilized stands during peak summer. Even if the effect is several times lower on an annual basis, the results still suggest that nitrogen fertilization can have a much stronger influence on net ecosystem production than can be expected from a direct stimulation of primary production alone.

During the last decade it has been debated to which extent nitrogen (N) fertilization of forest ecosystems can mitigate climate change by increasing carbon (C) sequestration. The debate was intensified in 2007 when Magnani *et al*.^1^ suggested that net ecosystem production (NEP) was strongly related to N deposition. More remarkably, the slope of the relationship suggested that up to several hundred kg C could be sequestered per kg N of total deposition. The results was criticized on, among other things, the ground that such high C:N ratios in reality is ecologically implausible given the C:N stoichiometry of forest ecosystem compartments^2^. More conservative estimates suggest that the ratio between N deposition and C sequestration is the range of 30–75 kg C per kg N^2^,^3^,^4^, all the way down to 16 kg C per kg N^5^.

The controversy over the effect of N fertilization on C sequestration and NEP has mainly focussed on the effect of N fertilization on primary production. Increased NEP in forest ecosystems in response to N fertilization can, however, not be attributed to increased primary production alone. There is also ample evidence that N fertilization results in significantly reduced soil respiration rates that cannot be fully accounted for by decreased root respiration^6^,^7^,^8^. In fact, it appears as if N fertilization has a strong direct effect on the decomposition rate of soil organic matter (SOM)^9^,^10^,^11^, which could partly explain observations of strong increases in NEP in response to N fertilization.

It is not well understood why N fertilization commonly result in decreased SOM decomposition, but several hypothesis have been proposed. N-fertilization causes shifts in the microbial community composition^12^, which could potentially result in slower decomposition rates^13^. It has also been suggested that fertilization with N may result in condensation reactions that stabilize SOM and render it less vulnerable to enzymatic attack^10^,^14^. Finally, N-fertilization might result in decreased “N mining” by ectomycorrhizal fungi and other microorganisms, i.e. the microbes do not have to decompose SOM to the same extent in order to meet their N demand^15^,^16^. Even if the hypotheses above are appealing, conclusive evidence regarding their quantitative importance for the reduced decomposition is still lacking^7^,^10^.

An unexplored explanation to decreased SOM decomposition in response to N fertilization is that the fertilization results in decreased priming. Priming is a process where inputs of labile C compounds, e.g. root exudates, cause a change in the turnover rate of SOM^17^,^18^. The process appears to be one of the quantitatively most important for the decomposition of SOM, but the effect is highly variable and its mechanism is not well understood^19^,^20^,^21^. The extent of priming seems to be partly determined by the root exudation rate^22^,^23^ and a possible explanation to the variable priming is that the amount of C allocated belowground varies among various plant species, type of soil and also age of the plant^24^,^25^. Furthermore, since N fertilization may cause plant physiological responses that influence the proportion of fixed C that is allocated to roots and root exudates^26^,^27^,^28^, it is not unreasonable to suspect that decreased allocation of plant C belowground in response to N fertilization might result in decreased priming of SOM decomposition. Fertilization with N might also influence priming directly, as it has been suggested that priming is a distinct N mining response of the microbial community^15^,^16^. The picture is, however, complicated by the fact that the increased N availability has co-occurred with an increase in the atmospheric CO_2_ concentration. Elevated CO_2_ concentrations commonly result in increased allocation of plant C to root exudates^27^,^28^, and increased decomposition of SOM^19^. The combined effect of elevated CO_2_ and increased N availability on priming of SOM decomposition remains uncertain.

The aim of this study was to test the hypothesis that decreased SOM decomposition in response to N fertilization can be explained by diminishing priming effects, and to determine to which extent priming of SOM decomposition is manifested as C or N mineralization under different loadings of labile C and N. We also aimed at determining whether any changes that occurs in response to N fertilization is a long-term effect dependent on a shift in microbial community composition (N fertilization in field), or an immediate effect caused by increased N availability and decreased N mining (N addition in lab at the start of the experiment). Finally, we aimed to determine whether different microbial C sinks (fungi and bacteria) had a different effect on the decomposition of SOM. The experiment was conducted by testing how gross N mineralization and respiration of SOM responded to glucose additions in N fertilized and pristine nemoral/nemo-boreal spruce forest soils. We show that increased input of labile C caused a decoupling of soil C and N cycling, manifested as a significant reduction in respiration of SOM that coincided with a substantial increase in gross N mineralization. The magnitude of the effect is dependent on the rate of labile C and N input, and can potentially result in an increase in NEP by up to 51 kg C ha^−1^ day^−1^ in N fertilized stands during peak summer. These findings suggest that the combined effects of elevated CO_2_ and N fertilization on belowground C and N cycling will result in much stronger effect on NEP than can be expected from a direct stimulation of primary production by atmospheric N deposition alone.

## Results

The total respiration, i.e. the sum of the respiration of added glucose and respiration of soil organic matter (SOM), differed between treatments (p < 0.001, ANOVA) and increased with increasing glucose concentration (p < 0.001, ANOVA) (Tables 1 and 2). For any given level of glucose addition, total respiration was highest in soil from N fertilized forest stands with a high density of trees, but the difference was not strong enough to be significant compared to control stands (p > 0.05, Tukey HSD). In contrast, total respiration in N fertilized stands with the same density of trees as control stands was lower than that in the control stands (p < 0.001, Tukey HSD), at all levels of glucose addition (Table 1). When soil collected from the control stands received inorganic N in the lab, we found no significant difference in the respiration rate compared to the *in situ* N fertilization treatment with the same density of trees (p > 0.05, Tukey HSD). Thus, the immediate response to N additions in the lab was similar to that caused by N fertilization *in situ* (Table 1). The increase in respiration in response to glucose additions was significant at all glucose concentrations (p < 0.01 to p < 0.001, Tukey HSD) except the lowest (20 mg glucose per kg soil).

Addition of glucose enriched with ^13^C allowed us to partition the total respiration into respiration of glucose and respiration of native SOM. This resulted in the emergence of a different pattern to that of the total respiration. Contrary to our hypothesis glucose additions generally resulted in negative priming (Table 1), i.e. decreased respiration of SOM (Suppl. Fig. 1). The effect was obvious already 4 hours after glucose addition and increased with increasing glucose concentration (p < 0.001, ANOVA, Table 2). The magnitude of the response to glucose additions differed between the different treatments, as indicated by the significant (p < 0.05) interaction between glucose and treatment (Table 2). The strongest decrease in respiration of SOM was found in N fertilized forest stands with a high density of trees, which differed significantly from the control treatment (p < 0.05, Tukey HSD) and the other *in situ* N fertilization treatment (p < 0.001, Tukey HSD). In contrast, the inhibitory effect of glucose additions on respiration of SOM (i.e. the negative priming effect) did not differ between the control treatment and the *in situ* N fertilization treatment with the same density of trees as the control (p < 0.05, Tukey HSD). When the priming effects of glucose additions described above was accounted for, we found that the cumulative respiration of SOM was generally lower in the two *in situ* N fertilization treatments compared to the control (Table 3, Suppl. Fig. 1). A rough estimation of the magnitude of the effect at the stand level, suggest that the combined effect of N fertilization and labile C inputs resulted in a reduction in respiration of SOM by between 7–13 kg CO_2_-C ha^−1^ day^−1^ in the N-fertilization treatment with the same density of trees as the control, and by between 0–7 kg CO_2_-C ha^−1^ day^−1^ in the N-fertilization treatment with high density of trees (Table 3).

In contrast, glucose additions generally primed the mineralization of N in all treatments, resulting in a strong increase in gross N mineralization that was most pronounced at high glucose concentrations (p < 0.001, ANOVA) (Tables 1 and 2). The increase was significant already at the lowest concentration of glucose (p < 0.01, Tukey HSD), but the magnitude of the increase varied among treatments (p < 0.001, ANOVA, Table 2). In N-fertilized forest stands with high density of trees gross N mineralization more than doubled at the lowest glucose addition, while it increased by more than 10-fold at the highest glucose concentration (Table 1). The effect was less pronounced in control soil and in soil from N fertilized forest stands with a tree density corresponding to that of the control, but there was still a 10 to > 500% increase in gross N mineralization, depending on the glucose concentration. In contrast, inorganic N addition in the lab to soil samples collected from the control plots seemed to reduce the gross mineralization rate (p < 0.001, Tukey HSD) and also resulted in a less pronounced priming effect in response to glucose additions (Table 1). When the above described effects of glucose additions on gross N mineralization was accounted for, we found that gross N mineralization was generally higher in the *in situ* N fertilization treatments with high density of trees compared to the control treatment at corresponding concentration of glucose, while the same was not the case in the *in situ* N fertilization treatments with the same density of trees as the control (Table 3). A rough estimation of the magnitude of the effect at the stand level suggest that gross N mineralization in the organic horizon increased by between 2–14 kg N ha^−1^ day^−1^ in response to the combined effect of N fertilization and labile C inputs in the N-fertilized treatment with high density of trees (Table 3).

Taken together, the results above suggest that glucose additions and N fertilization resulted in decoupling of SOM respiration and gross N mineralization, possibly caused by preferential use of glucose as a C and energy source by the microbial community. The decoupling generally resulted in decreased respiration of SOM and increased mineralization of N, and was most pronounced in the N-fertilized plot with high tree density (Table 1). A rough estimation of the magnitude of the effect at the stand level suggest that the net effect of the decoupling can result in an increase in NEP by between 3–20 kg C ha^−1^ day^−1^ (in the N fertilization treatment with the same density of trees as the control) and by between 10–51 kg C ha^−1^ day^−1^ (in the N fertilization treatment with high density of trees), relative to the control treatment (Table 3).

**Figure 1:**
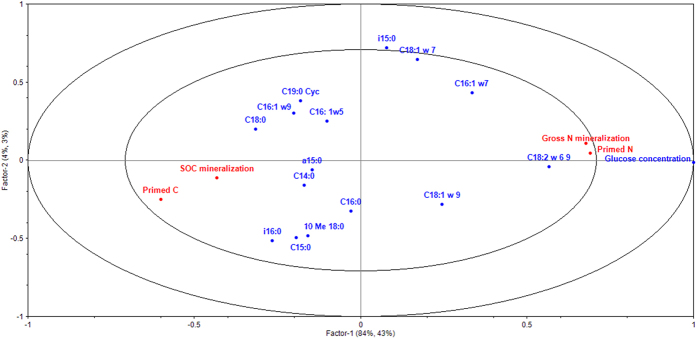
PLSR-analysis of the dependency of respiration of SOM, primed C, gross N mineralization, and primed N on labile C. High concentrations of labile C (glucose) resulted in high incorporation of glucose derived ^13^C in the fungal biomarker PLFA 18:2*ω*6,9, increased N priming and gross N mineralization, and negative C priming resulting in decreased respiration of SOM.

In order to identify the microbial groups responsible for the observed patterns in SOM respiration and gross N mineralization, a PLSR-analysis was performed. The glucose concentration and the proportion of glucose derived ^13^C-that was recovered in different PLFA’s was used as predictors, while respiration of SOM, primed C, gross N mineralization, and primed N were defined as response variables. The first factor on the correlation loadings plot explained the majority of the variation in the data (Fig. 1, R^2^ = 0.88, Q^2^ = 0.46), where R^2^ represents the variability of the responses explained by the model and Q^2^ represents a cross-validated R^2^ (leave-one-out method), i.e. the amount of variation in the *y*-variable that can be predicted by the model. According to the PLSR analysis, primed C and respiration of SOM were negatively correlated to primed N and gross N mineralization (Fig. 1). Glucose concentration was the main driver of the observed results, with high concentrations leading to high gross N mineralization and N priming, and at the same time decreased respiration of SOM and negative C priming (Fig. 1). Fungi appear to be mediating this response, since there was an increase in the proportion of glucose derived ^13^C that was recovered in the fungal biomarker PLFA 18:2*ω*6,9^29^ at high glucose concentrations (Fig. 1).

## Discussion

The extent to which forest ecosystems act as atmospheric C sinks or sources is determined by the net ecosystem production (NEP), i.e. the difference between gross primary production and ecosystem respiration. Elevated atmospheric CO_2_ concentrations commonly result in at least a temporary stimulation of primary production^30^,^31^, but also in increased allocation of plant C to root exudates^27^,^28^. Labile C compounds in these exudates can ‘prime’ the decomposition of soil organic matter (SOM)^19^,^22^,^32^. It has, therefore, been suggested that elevated atmospheric CO_2_ concentrations can result in increased decomposition of SOM and decreased C stocks in forest soils^33^, constituting a positive feedback to climate change. Our results contradict this view. In fact, labile C input inhibited rather than stimulated respiration of SOM. The inhibitory effect was most pronounced in dense N-fertilized stands, but respiration of SOM was also lower than in control stands in the less dense N fertilized stands. These findings suggest that N fertilization in combination with input of labile C can increase NEP not only by stimulating primary production, but also by decreasing decomposition and respiration of SOM. The strength of the effect is not negligible. In fact, our results suggest that N fertilization combined with labile C input has the potential to reduce respiration of SOM with as much as to 13 kg CO_2_-C ha^−1^ day^−1^ in the organic horizon alone at peak summer temperatures, even if more than two years had passed since the fertilization. The decrease in respiration is in accordance with observations from the Duke Free-Air CO_2_ enrichment experiment, where Billings and Ziegler^34^ found elevated CO_2_ in combination with N fertilization to result a 28% reduction in heterotrophic soil respiration compared to the other treatments, while the total soil CO_2_ efflux decreased with 21% in response to N fertilization at the same site^35^.

The experimental design do not allow us to conclude the reason for the observed results, but a possible explanation is that N fertilization has decreased plant C allocation to root exudates, resulting in a shift in the microbial community composition to a community that is less adapted to use exuded C to prime the decomposition of SOM. However, the observation that the respiration of SOM tended to decrease in a similar manner in response to N fertilization, regardless if the N fertilization was done *in situ* or *in vitro* just before the start of the experiment, speaks against this conclusion. This suggests that the decreased respiration of SOM was not a long-term effect dependent on a shift in microbial community composition^12^,^13^, but rather an immediate effect caused by increased N availability.

The decrease in respiration of SOM in response to labile C inputs that occurred in all treatments can be explained by preferential substrate use by the soil microbial community^36^. That is, when the soil microorganisms were provided with labile C, they decreased their decomposition and respiration of more recalcitrant SOM. Microbial immobilization of labile C can also directly contribute to increased soil C sequestration^37^. However, the extent to which root exudates and other labile C sources immobilized in the microbial biomass remains in soil depends on which microbial groups the labile C is immobilized in. For example, fungal residues are less decomposable and have longer turnover times compare to bacterial residues, and soil microbial communities dominated by fungi seem to retain more C in soil^38^,^39^. A high immobilization of labile C in fungi rather than bacteria can, therefore, be expected to make a stronger contribution to long-term C storage. In this study the respiration of SOM was negatively correlated to the proportion of glucose derived ^13^C that was incorporated into fungi. The response was stronger at high glucose concentrations, suggesting that high glucose concentrations resulted in high incorporation of glucose derived C into fungi and strong inhibition of the respiration of SOM. It, therefore, seems as if increased root exudation of labile C in response to elevated CO_2_^27^,^28^ may under some conditions result in increased fungal immobilization of the exuded C, decreased respiration of SOM, and in the long term, increased NEP and SOM stocks. Accordingly, Clemmensen *et al*.^40^ recently demonstrated that belowground input of plant C significantly contributes to SOM formation, possibly by plant C allocation to root associated fungi. In our study the negative priming induced by labile C input was most pronounced in the N fertilized treatment with a high density of trees, while there was no obvious difference in the priming response between control stands and N fertilized stands with the same density of trees as control stands. This suggests that the belowground C-cycling response to labile C input is partly dependent on the density of trees. It was beyond the scope of this study to provide a mechanistic explanation to why this is the case, but a possible reason is that there is a higher fungal abundance in the treatment with a high density of trees, as indicated by the negative relationship between ^13^C incorporation into fungi and primed C found in the PLSR-analysis.

The preferential use of labile C by fungi was accompanied by selective ‘mining’ of SOM for N, resulting in a strong increase in gross N mineralization. The opposite relationship was found between ^13^C incorporation into bacterial biomarker PLFAs, respiration of SOM and gross N mineralization, suggesting that fungi and bacteria responds differently to input of labile C. The strong priming of gross N mineralization by labile C inputs also infers that there was a massive increase in the release of plant available N, and supports previous suggestions that priming is a distinct N-mining response of the microbial community^41^. The findings also explain recent observations that trees exposed to elevated CO_2_ can acquire additional soil N by increasing their belowground C allocation, even in N-limited ecosystems^42^,^43^,^44^.

Priming of N mineralization by labile C input was, again, most pronounced in the dense N-fertilized stand. That is, not only was the decrease in respiration of SOM in response to labile C input most pronounced in this treatment, but it also had the highest increase in the production of the most commonly limiting plant nutrient. Interestingly, there was no obvious difference between control stands and N fertilized stands with the same density of trees as control stands. This suggests that the belowground C- and N-cycling response to N fertilization is partly ameliorated by the density of trees. The data at hand only allows us to speculate on why this was the case. In addition to the possible influence of the treatments on the abundance of fungi discussed above, another possible explanation is that the concentration of directly available N differed among the N fertilization treatments. Accordingly, the lowest increase in gross N mineralization in response to input of labile C was found in the treatment that received inorganic N *in vitro* at the start of the experiment, suggesting that the increased microbial N demand induced by input of labile C could partly be satisfied by direct uptake of available N. Labile C additions caused a much stronger increase in gross N mineralization in the *in situ* N fertilization treatment with low density of trees, while the strongest increase was found in the *in situ* N fertilization treatment with high density of trees. It is possible that the high density of trees have resulted in higher tree uptake of the fertilizer in the latter treatment. As a consequence, the microbial community might have been more reliant on mineralizing labile organic N compounds in order to meet an increased N demand induced by the glucose additions.

Only a limited number of other studies have examined the influence of trees and tree roots on *in situ* gross N mineralization rates. Bengtson *et al*.^45^ reported an average gross N mineralization rate of 7.5 kg N ha^−1^ day^−1^in a Swedish beech/oak forest, i.e. in the same range as the here reported N mineralization rates. The N mineralization exhibited a strong spatial variation at the meter scale, which could partly be explained by the influence of trees^46^. Likewize, Holz *et al*.^47^ found *in situ* gross N mineralization rates to vary between 1.3 to 4.5 mg N kg^−1^ day^−1^ in a Swedish spruce forest, with the slowest rate found in a treatment where roots and ectomycorrhiza was excluded. When recalculated to kg N ha^−1^, using the same conversion factor as in this manuscript, this corresponds to between 1.3–4.5 kg N ha^−1^ day^−1^. Combined, these studies support our observation that tree roots and root associated fungi strongly regulate gross N mineralization rates.

Even if the response of gross N mineralization to labile C additions varied among treatments, an analysis of the combined effect of N fertilization and labile C input on gross N mineralization and respiration of SOM suggest that NEP strongly increased in both *in situ* N fertilization treatments. A simultaneous increase in C sequestration in plant biomass due to higher N mineralization rates, combined with decreased respiration of SOM, suggests that N fertilization in combination with labile C input can have a much stronger positive effect on NEP than what would be expected from stimulation of primary production by N fertilization alone. If added on top of the C that is sequestered in tree biomass as a direct response to the fertilization, these findings may partly explain why N fertilization can induce an increase in NEP that is high enough to seem implausible given the C:N stoichiometry of primary producers^1^.

The observed decrease in respiration of SOM in response to labile C input should not be interpreted as a lack of positive priming or equated to decreased decomposition of SOM. The initial stages in the decomposition of humus, of lignin and other polyaromatic compounds are largely governed by oxidative rather than hydrolytic enzymes, or in the case of brownrot and ectomycorrhizal fungi, by unspecific Fenton based hydroxyl radical production^48^. The result is a release of compounds that can be further decomposed by hydrolytic enzymes, followed by microbial uptake and partitioning of different elements into anabolic and catabolic processes. Stoichiometric variations in the nutrient status of soil microorganisms will determine to what extent the decomposition is manifested as C and N mineralization^49^,^50^. In this chain of events the oxidative decomposition of SOM appears to be the rate limiting step^51^,^52^. In this study labile C input resulted in a strong increase in gross N mineralization that must have been preceded by decomposition of SOM into bioavailable N containing organic compounds vulnerable to enzymatic attack. This suggests that labile C inputs might have caused priming of SOM decomposition even if no increase in C mineralization occurred. These findings suggest that priming is of minor importance for the mineralization of SOM derived compounds, since it rather stimulates the oxidative degradation of SOM into compounds available for microbial uptake and mineralization. Even so, the importance of priming for SOM decomposition has almost exclusively been quantified by measuring how respiration of SOM responds to input of labile C. In other words, decomposition of SOM is usually equated to soil C mineralization rates, which could potentially result in erroneous conclusions about the importance of priming for SOM decomposition, C sequestration and NEP under different scenarios of environmental change.

In conclusion, the results show that N fertilization and labile C inputs can increase NEP and soil C storage by inhibiting microbial respiration of SOM, by contributing to the build-up of SOM derived from fungal biomass, and by increasing the production of plant available N. The results are in agreement with a recent analysis of 19 datasets obtained from the Duke Free-Air CO_2_ enrichment experiment, which suggest that elevated CO_2_ results in increased plant N uptake and decreased turnover of SOM^44^. These findings have implications for predictions of how NEP and soil C stocks will respond to the combination of elevated CO_2_ and N fertilization. A rough estimate of the combined effect of N fertilization and labile C inputs found in this study suggest that N fertilization can result in a potential increase in NEP of up to 51 kg C ha^−1^ day^−1^, based on numbers from the organic horizon alone. The increase occurred even if the samples were collected more than two years after the time of fertilization, and should be added on top of the C that is sequestered in plant biomass as direct result of the fertilization. The estimated increase in NEP does not include the formation of SOM caused by fungal immobilization of the labile C, which will further contribute to NEP^38^,^39^,^40^. However, due to the limited spatial and temporal resolution of our experiment, the suggested potential increase in NEP of up to 51 kg C ha^−1^ day^−1^ should be interpreted with caution. Since the experiment was carried out at 20 °C and the annual average temperature at the site is 3.8 °C, the response is likely to be several times lower on an annual basis. It is also likely that the realized increase in NEP varies among ecosystems, which calls for further experiments at a larger spatial resolution that includes seasonal and annual variation in temperature, precipitation, light, duration of growth season, etc. Even so, the findings still suggest that the combined effects of elevated CO_2_ and N fertilization on belowground C and N cycling will result in much stronger effect on NEP than can be expected from a direct stimulation of primary production by atmospheric N deposition alone.

## Material and Methods

### Soil sampling

Soil samples were collected in November 2013 from the organic horizon in a Norway Spruce forest located in the nemoral/nemo-boreal zone in South Sweden at Tönnersjöheden Research Park (in the county of Halland, lat. 56°41′–42′ long. 13°5′–7′), which has an average annual temperature of 3.8 °C. There were three replicate plots (measuring 30–40 m × 25 m) of each treatment, which included control (C), N fertilization (N) and N fertilization in dense forest stands (ND). The stand was a 32 year old (at the time of fertilization) Norway spruce forest that was thinned to a density of approximately 940 stems ha^−1^ (winter 2010/2011), except for the dense stand which has approximately 1500 stems ha^−1^, growing on a podzol with an organic horizon with a depth ranging between 3–16 cm^53^. Nitrogen was applied in July 2011 as single dose of 200 kg N ha^−1^ (as ammonium nitrate). We also included a treatment (CN) where soil collected from the control plots (C) received inorganic N in the lab at approximately the same level as in the N fertilized plots (145 mg N kg). Soil was collected from five randomly selected locations within each replicate plot using a soil corer with a diameter of 5 cm. The organic horizon soil in the five cores was separated from the mineral soil, pooled, and stored on ice during the transport back to the lab. Upon arrival in the lab, roots were removed by hand, after which the soil was sieved (4 mm) and stored at 4 °C until the start of the experiment (<48 hours), which was conducted at ambient temperature (20 °C). The organic matter content and gravimetric water content of the collected soil samples was 0.85 g OM g^−1^ soil (estimated by loss on ignition) and 0.27 g H_2_O g^−1^ soil, respectively, and did not differ among treatments.

### Soil respiration and potential priming

Soil respiration and potential priming was estimated by weighing field moist soil (10 g) from each replicate plot into five separate 120 ml serum bottles. The bottles then received 1.0 ml of either distilled water or a ^13^C-labelled glucose solution (6.0 atom% ^13^C) at concentrations of 20, 80, 320 or 1280 mg glucose kg^−1^ soil. The soil was thoroughly mixed and the bottles were flushed with standardized air and sealed with a rubber septa and a crimp cap immediately after the additions. The respiration rate and potential priming was estimated 4 and 24 hours after the glucose addition by transferring 1.0 ml of the headspace of the serum bottles to 12 ml He-flushed Exetainers (Labco, UK) using a Hamilton GASTIGHT^®^ syringe. The concentration and ^13^C/^12^C ratio of CO_2_ in the Exetainers were measured on a GasBench II connected to a Delta V Plus isotope-ratio mass spectrometer (Thermo Scientific Inc., Bremen Germany), at the stable isotope facility, Department of Biology, Lund University.

The fraction of respired CO_2_ in the headspace of the serum bottles that originated from glucose and SOM, respectively) was calculated using a two end-member isotopic mixing model (Eqs 1 and 2):


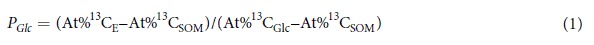



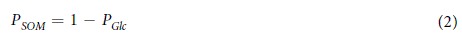


Where *P*_*Glc*_ and *P*_*SOM*_ is the proportion of CO_2_ derived from respiration of glucose and SOM, respectively, At%^13^C_E_ is the measured atom% ^13^C of respired CO_2_ in soils that received glucose, At%^13^C_SOM_ is the atom% ^13^C of respired CO_2_ in control soils that did not receive glucose, and At%^13^C_Glc_ is the atom% ^13^C of the added glucose.

The potential priming was calculated by subtracting the respiration of SOM in samples that did not receive glucose from the respiration in samples from the corresponding treatment that received glucose. Since the respired CO_2_ in samples that did not receive glucose originates from SOM, divergence from this rate indicates a change in respiration of SOM induced by the glucose additions.

### Gross N mineralization

The gross N mineralization rate at 4 and 24 hours after glucose addition was estimated using the ^15^N-pool dilution technique. Briefly, field moist soil (10 g) from each replicate plot in the different treatments was placed in 15 separate 100 ml plastic container. The containers then received either distilled water or glucose at concentrations mentioned above (20, 80, 320 or 1280 mg glucose kg^−1^ soil), in combination with 0.5 ml of a ^15^NH_4_Cl solution (99 atom% ^15^N, 3.2 mg N kg^−1^ soil). The soil was thoroughly mixed and the containers were sealed with a tight fitting cap. Inorganic nitrogen was extracted from one set of the triplicate beakers immediately after addition of the ^15^NH_4_Cl-solution, from the second set 4 hours after the addition, and from the third set 24 hours after the addition.

Inorganic N was extracted by adding 50 ml of 1 M KCl (Merck, Darmstadt, Germany) to each beaker. The beakers were put on a horizontal shaker for an hour, after which the extract was filtered through a Whatman GF/F-filter. The filtrate was collected in a new set of beakers and NH_4_^+^ isolated from the filtrate, using standard diffusion procedures^54^, by adding an acid trap followed by 0.2 g MgO. The beakers were closed and shaken for approximately 72 hours on a horizontal shaker (100 rpm), after with the traps were removed and the filter discs dried in a desiccator. The dried filter discs were placed in tin cups and analyzed for ^15^N/^14^N concentrations at the stable isotope facility at the Department of Biology, Lund University. Samples were flash-combusted in a Flash 2000 elemental analyzer (Thermo Scientific Inc., Bremen Germany) and the total amount of N in the samples determined using the elemental analyzer’s thermal conductivity detector. The isotopic ratio was determined on a Delta V Plus isotope-ratio mass spectrometer connected to the elemental analyzer via the ConFlow IV interface (Thermo Scientific Inc., Bremen Germany). The gross N mineralization rate was calculated from the differences in concentration and ^15^N content of NH_4_^+^ between the samples taken immediately, 4 h and 24 h after the addition of the label, using the ^15^N pool dilution technique previously described in Bengtson *et al*.^46^. The calculations assume constant gross mineralization rates and that no ^15^N is recycled to the enriched pool (in this case NH_4_^+^) during the measured period. The short assay (24 h) was an effort to meet this assumption. Primed N was calculated by subtracting the gross N mineralization in samples that did not receive glucose from the gross N mineralization in samples from the corresponding treatment that received glucose.

### PLFA extraction and analysis

PLFA’s was extracted from 1 g of soil 4 and 24 hrs after the addition of glucose, using a modified Bligh and Dyer cold liquid-liquid extraction procedure^29^,^55^. Briefly, soil samples were vortexed for 15 s in a 1:2:0.8 (v/v/v) (CHCl_3_: MeOH: citrate buffer) solution and then centrifuged at 5000 rpm for 10 min. The supernatant was transferred to a large test tube. The same extraction and centrifugation procedure was carried out on the remaining pellet (using 5 ml of Bligh and Dyer solution) and the two supernatants combined. The supernatant was then split into two phases by adding equal amount of (4 ml) CHCl_3_ and citrate buffer, after which the samples were left overnight. The following day the lower phase was transferred to a test tube and dried down under gentle stream of nitrogen at 40 °C. The dried down soil lipid extract were then dissolved with CHCl_3_ and transferred to a silica based sorbent cartridges (Bond Elut^®^ LRC-SI, Agilent Technologies, U.S.A). Lipids were eluted from the cartridges using chloroform, acetone and methanol and the latter eluate, containing the PLFAs, was collected and subjected to mild alkaline methanolysis at 37 °C for 15 min in a temperature controlled water bath. The fatty acid C19:0 was used as internal standard spiked into each sample before the esterification. The chromatographic separation and ^13^C isotopic analysis of fatty acid methyl esters (FAME) were carried out using a Thermo Trace GC Ultra connected to a Delta V Plus isotope-ratio mass spectrometer via a GC Isolink II preparation device and ConFlow IV interface (Thermo Scientific Inc., Bremen Germany). The GC was equipped with a HP-5MS UI column (60 m × 0.25 mm I.D., 0.25 μm thick stationary phase, Hewlett Packard^®^). The inlet port temperature was set at 250 °C and the flow of Helium carrier gas at 1.5 ml min^−1^. The operating condition of the GC was as follows: splitless injection with initial temperature of 50 °C held for 1 min; ramped at 15 °C min^−1^ to 160 °C; followed by ramping at 2 °C min^−1^ to 200 °C held for 10 min; ramping to 230 °C at 3 °C min^−1^ and ramping further at 20 °C min^−1^ to the final temperature of 300 °C and held for 4 min. This ramping program resulted in good separation between different FAME isomers.

### Calculation of C and N effects on net ecosystem production

The effect of N fertilization on net ecosystem production (NEP) at different input rates of labile C was estimated using eq. (3):


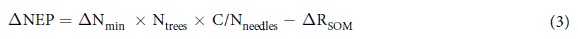


Where ΔNEP represent the difference in NEP between the N fertilized treatments and the control at a certain concentration of glucose, ΔN_min_ the difference in gross N mineralization between the N fertilized treatments and the control at a certain concentration of glucose, N_trees_ the fraction of the mineralized N that is taken up by trees, C/N_needles_ the C/N ratio of the spruce needles, and ΔR_SOM_ the difference in respiration of SOM between the N fertilized treatments and the control at a certain concentration of glucose.

Two of the variables in equation 3, N_trees_ and C/N_needles_, were based on literature values. The percentage N in spruce needles found in an N fertilization study by Gundale *et al*.^5^ varied between 1.16–1.45%, which based on a C content of 50% gives a C/N ratio of between 34–43. In the same study between 7–9% of the added N fertilizer were recovered in trees. In order to account for this variation, as well as for the variation in the measured variables, we based our analysis of the combined effect of N fertilization and labile C input on a probabilistic Monte-Carlo analysis, rather than on average values. The analysis was performed in @RISK 5.7 (Palisade corporation, Ithaca, NY, USA) by sampling the probability distribution of the measured variables in Equation 3 (i.e. gross N mineralization and respiration of SOM in the control and treatment at a certain glucose concentration), as well as N_trees_ and C/N_needles_, using the Latin Hypercube technique. C/N_needles_ were set to vary between 34–43 and N_trees_ between 0.07–0.09, thus covering the full range of values found in the Gundale *et al*. study. The variation in gross N mineralization and respiration of SOM was accounted for by using the mean and normal probability distribution (estimated from the average coefficient of variation of the measurements) as input. These probability distributions were truncated at the 95% upper and lower confidence limits. In total 200,000 simulations were performed, followed by a sensitivity analysis aimed at identifying the input distributions with the strongest contribution to the variation in the output distribution. The sensitivity analysis was performed using an in-built based on multiple regression analysis and demonstrated that that the results were mainly dependent on the measured variables, rather than on the assumed variables (data not shown). All rates were recalculated to kg ha^−1^ d^−1^ prior to the analysis, based on an estimated mass of the organic horizon of 1000 tons ha^−1^.

### Statistics

Differences in total respiration, priming and gross N mineralization among the different treatments and glucose concentrations were tested by a factorial ANOVA followed by Tukey’s HSD test. Total respiration and gross N mineralization data were log10 transformed prior to analysis to ensure homogeneity of variance and normal distribution of residuals. The analyses were performed using STATISTICA version 12 (StatSoft Inc, Tulsa, OK, USA). Differences in respiration of SOM, gross N mineralization and NEP between the two *in situ* N fertilization treatments and the control, at different input rates of labile C, were further assessed by testing if ΔR_SOM_ (the difference in respiration of SOM between the N fertilized treatments and the control at a certain concentration of glucose), ΔN_min_ (the difference in gross N mineralization between the N fertilized treatments and the control at a certain concentration of glucose), and ΔNEP (the difference in NEP between the N fertilized treatments and the control at a certain concentration of glucose) differed significantly from zero. The results were considered to be significant if the upper or lower 95% confidence limit of the probability distributions produced by 200,000 Monte Carlo simulations (described above) of ΔR_SOM_, ΔN_min_ and ΔNEP did not overlap with zero. In order to identify correlations between the investigated variables and identify the microbial groups responsible for the observed patterns in priming and C and N mineralization a PLSR-analysis was performed using Unscrambler X^®^ Ver. 10.3 statistical software (CAMO Inc., Oslo, Norway). PLSR is an extension of multiple linear regression without the limitations that co-linearity and inter-correlation can impose on extracting the correct structural information from x and y variables^56^,^57^.

## Additional Information

**How to cite this article:** Ehtesham, E. and Bengtson, P. Decoupling of soil carbon and nitrogen turnover partly explains increased net ecosystem production in response to nitrogen fertilization. *Sci. Rep.* **7**, 46286; doi: 10.1038/srep46286 (2017).

**Publisher's note:** Springer Nature remains neutral with regard to jurisdictional claims in published maps and institutional affiliations.

## Supplementary Material

Supplementary Figure 1

## Figures and Tables

**Table 1:** The effect of N fertilization and labile C input on cumulative respiration, priming and gross N mineralization 4 and 24 hours after addition of glucose.

Treatment	Glucose added	Total respiration				Priming				Gross N mineralization			
	(mg C kg^−1^)	(mg C kg^−1^)				(mg C kg^−1^)				(mg N kg^−1^)			
		4 h		24 h		4 h		24 h		4 h		24 h	
C	0	13.1	(2.7)	68.9	(14.7)					0.54	(0.06)	4.01	(0.47)
	20	14.0	(2.9)	68.6	(15.3)	−0.24	(0.20)	−1.69	(0.24)	1.28	(0.29)	6.53	(1.96)
	80	16.3	(2.8)	69.7	(13.5)	−1.22	(0.58)	−5.41	(1.02)	1.84	(0.51)	6.93	(1.22)
	320	20.7	(3.2)	88.0	(13.1)	−1.31	(0.61)	−7.30	(3.76)	2.29	(0.77)	9.82	(1.64)
	1280	31.7	(7.2)	149.1	(21.5)	−0.77	(0.49)	−9.85	(2.27)	2.89	(1.18)	20.49	(4.94)
CN	0	13.2	(2.7)	61.9	(14.4)					0.33	(0.10)	2.51	(0.75)
	20	13.6	(2.8)	63.1	(13.4)	−0.61	(0.21)	0.67	(0.24)	0.67	(0.28)	2.03	(0.33)
	80	15.7	(2.8)	67.8	(12.8)	−1.39	(0.44)	0.14	(1.18)	1.35	(0.43)	1.64	(0.32)
	320	19.7	(3.7)	88.4	(13.5)	−1.51	(0.42)	−2.90	(4.23)	1.01	(0.38)	3.40	(0.81)
	1280	23.4	(2.7)	130.4	(15.5)	−2.80	(0.56)	−13.16	(2.90)	3.05	(1.21)	6.91	(2.05)
N	0	10.8	(0.4)	53.5	(1.6)					0.57	(0.14)	4.29	(1.04)
	20	12.3	(0.4)	58.4	(1.5)	0.40	(0.03)	3.04	(0.05)	1.42	(0.07)	5.22	(0.90)
	80	14.7	(0.0)	58.9	(0.6)	−0.26	(0.03)	−0.41	(0.13)	1.68	(0.09)	6.34	(0.95)
	320	20.5	(0.3)	80.3	(0.7)	−0.21	(0.13)	0.67	(0.60)	2.20	(0.42)	8.51	(1.60)
	1280	24.4	(0.8)	133.9	(4.5)	−1.44	(0.31)	−7.21	(1.23)	4.80	(2.40)	22.65	(6.89)
ND	0	13.9	(1.3)	73.4	(7.3)					0.41	(0.09)	3.09	(0.67)
	20	16.0	(1.6)	74.5	(7.8)	−1.04	(0.14)	−9.96	(0.17)	1.64	(0.31)	8.71	(1.51)
	80	19.2	(1.4)	79.6	(7.9)	−1.26	(0.43)	−9.81	(0.70)	1.85	(0.52)	11.61	(0.34)
	320	25.5	(3.3)	96.5	(6.7)	−1.64	(0.01)	−14.46	(2.60)	3.74	(0.71)	15.69	(2.61)
	1280	41.5	(4.7)	180.0	(14.0)	−1.92	(0.68)	−21.03	(4.01)	7.89	(3.33)	34.91	(2.46)

Treatments included control **(C)**, N fertilization **(N)** and N fertilization in dense forest stands **(ND)**. We also included a treatment **(CN)** where soil collected from the control plots **(C)** received inorganic N in the lab at approximately the same level as in the N fertilized plots. Values within brackets represent standard error of the mean (n = 3).

**Table 2:** ANOVA result from the test of the glucose addition and the different N fertilization treatments on total respiration (R_tot_), gross N mineralization N_min_, priming in the organic horizon of the investigated spruce forest.

		Treatment	Glucose	Treatment × Glucose
R_tot_	*F*	9.4	139.3	0.98
	*p*	**<0.001**	**<0.001**	0.48
Priming	*F*	8.2	15.1	2.01
	*p*	**<0.001**	**<0.001**	**<0.05**
N_min_	*F*	11.2	18.4	0.54
	*p*	**<0.001**	**<0.001**	0.88

Numbers represent *F*- and *p*-values, with significant *p*-values (*p* < 0.05) highlighted in bold.

**Table 3:** The combined effect of N fertilization and glucose addition on respiration of SOM, gross N mineralization and NEP in the organic horizon of the investigated spruce forest.

Treatment	Glucose added	ΔR_SOM_		ΔN_min_		ΔNEP	
	(mg C kg^−1^)	(kg C ha^−1^ day^−1^)		(kg N ha^−1^ day^−1^)		(kg C ha^−1^ day^−1^)	
N	20	−10.7	(3.1)*	−1.31	(0.30)*	6.6	(3.3)*
	80	−10.4	(2.9)*	−0.60	(0.33)*	8.6	(3.1)*
	320	−7.4	(2.9)*	−1.31	(0.45)*	3.4	(3.2)
	1280	−12.8	(2.7)*	2.16	(1.06)*	19.4	(4.3)*
ND	20	−3.8	(3.2)	2.18	(0.39)*	10.5	(3.5)*
	80	0.1	(3.1)	4.68	(0.53)*	14.4	(3.7)*
	320	−2.7	(2.9)	5.87	(0.71)*	20.8	(4.0)*
	1280	−6.7	(2.7)*	14.42	(1.59)*	51.1	(6.7)*

ΔR_SOM_ represent the difference in respiration of SOM between the N fertilized treatments and the control at a certain concentration of glucose, ΔN_min_ the difference in gross N mineralization between the N fertilized treatments and the control at a certain concentration of glucose, and ΔNEP the difference in C sequestration between the N fertilized treatments and the control at a certain concentration of glucose. All presented data are derived from the Monte-Carlo analysis, and results were considered to differ significantly from zero if the upper or lower 95% confidence limit derived from the analysis did not overlap with zero. Significant results are labelled with an asterix. Values represent the mean and standard deviation of 200,000 simulations.
