# Supplementary material for: Decoupling of soil carbon and nitrogen turnover partly explains increased net ecosystem production in response to nitrogen fertilization
Source: Sci Rep. 2017 Apr 13;7:46286. doi: 10.1038/srep46286 (PMC5390271; doi:10.1038/srep46286)
Supplement: Supplementary Figure 1 [file srep46286-s1.pdf]

# Decoupling of soil carbon and nitrogen turnover partly explains increased net ecosystem production in response to nitrogen fertilization

Emad Ehtesham[1,2], Per Bengtson[1*]

*[1] Department of Biology – Microbial Ecology, Lund University, Lund Sweden*
*[2] Now at: Department of Chemistry & Biotechnology - Swedish University of Agricultural Sciences, Uppsala, Sweden*

** Corresponding author: Department of Biology – Microbial Ecology, Lund University, Sölvegatan 37, 223 62 Lund. Tel. +46 (0) 46 2223760, E-mail: per.bengtson@biol.lu.se*

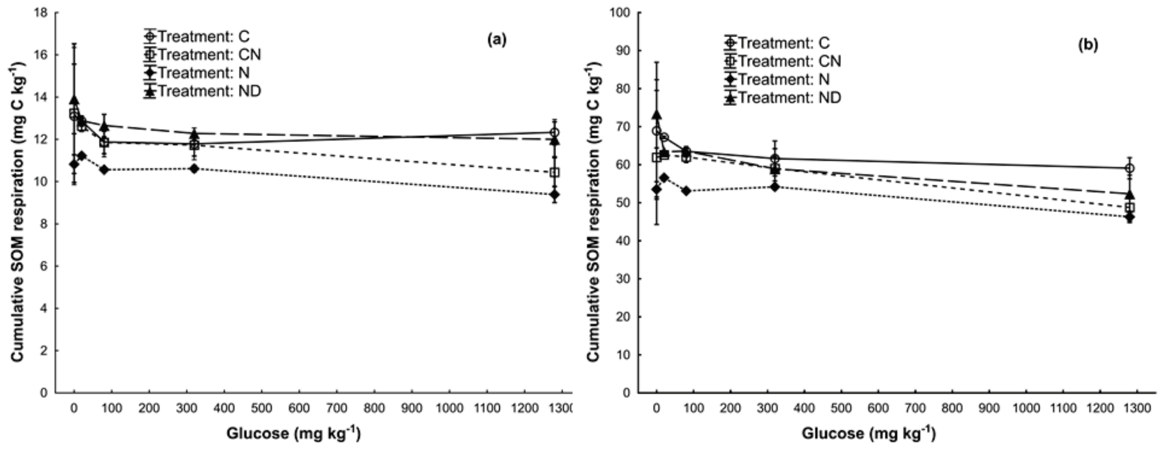


**Supplementary Figure 1. The effect of N fertilization and labile C input on cumulative respiration of SOM (a) 4 and (b) 24 hours after addition of glucose.** N fertilization and labile C (glucose) additions generally resulted in decreased respiration of SOM.
